# Examining the patient- and system-related forces influencing the responsiveness of hip fracture care in Zimbabwe: Findings from an ethnographic study

**DOI:** 10.1371/journal.pgph.0006631

**Published:** 2026-11-04

**Authors:** Nyasha Buwu, Kimberly Lakin, Rudo MS Chingono, Portia Mareke, Tendai Chiweshe, Cliff Zinyemba, Matthew Costa, Rashida A. Ferrand, Celia L. Gregson, Rachael Gooberman-Hill, Sarah Drew

**Affiliations:** 1 The Health Research Unit Zimbabwe, Biomedical Training and Research Institute, Zimbabwe; 2 Global Health and Ageing Research Unit, Bristol Medical School, University of Bristol, Bristol, United Kingdom; 3 Centre on Climate Change and Planetary Health, London School of Hygiene and Tropical Medicine, London, United Kingdom; 4 Oxford Trauma and Emergency Care, Nuffield Department of Orthopaedics, Rheumatology and Musculoskeletal Sciences, University of Oxford, Oxford, United Kingdom; 5 Department of Clinical Research, London School of Hygiene & Tropical Medicine, United Kingdom; 6 Bristol Medical School, University of Bristol, Bristol, United Kingdom; University of Global Health Equity, RWANDA

## Abstract

Rapidly rising longevity and the projected increase in fractures among older adults in Southern Africa underscores the necessity to strengthen healthcare systems in the region. Delivering orthopaedic care to older people within a multi-disciplinary framework is widely recommended. This ethnographic study sought to examine the responsiveness of hip fracture care in Zimbabwe and the influence of patient- and system-related forces We used ethnographic methods to examine the experiences of patients and caregivers and characterise hip fracture care provision. Case studies were undertaken with 40 patients (22 male, 18 female) and 33 caregivers (6 male, 27 female), including family members and friends. These data were complemented by interviews and observations of clinical practice with 42 healthcare professionals (23 female, 19 male) involved in the delivery of hip fracture services, including orthopaedic surgeons, physicians and physiotherapists. Participants were purposively sampled from six hospitals providing hip fracture care across Northern Zimbabwe. Snowball sampling was also used to recruit additional study participants. Data were analysed using an abductive analytical approach. Patients’ and caregivers’ experiences revealed lengthy hospital stays, poor communication concerning diagnosis and treatment, undignified continence management, and limited access to social support. Patients’ experiences highlighted the importance of access to pain relief medication, and the impact of unaffordable care – elements distinct from those within current responsiveness frameworks. Health professionals explained how resource constraints, understaffing, and changes in pain management processes impacted their ability to meet patients’ needs. Yet, their narratives told of the various actions they employed to circumvent health system constraints and respond to patient priorities. Considering these findings, we highlight key considerations for health systems responsiveness, including considering the role of socioeconomic factors. We also propose practical recommendations for improving responsive hip fracture care in Zimbabwe, such as developing guidelines for patient optimisation and standardisation of hip implant costs.

## Introduction

Populations in Southern Africa are rapidly ageing [1], due to a combination of improved public health services and population growth in the 20th century. Between 2019 and 2050, the United Nations predicts a 218% rise in the older populations in the region [2]. Generally reflecting the trend of other Southern African nations, Zimbabwe is currently undergoing a demographic transition. In 2013, an estimated 3.1% of the population was aged 65 years or older and, ten years later in 2023, this proportion had risen to 4.8% [3]. Ageing can result in several individual- and population-level challenges, including the rise of musculoskeletal conditions such as osteoporosis, which can lead to fragility fractures, including hip fractures. This occurs in part due to age-related bone loss, which makes bones more fragile and prone to breaking [4,5]. Globally, the incidence of hip fractures is predicted to increase from 1.66 million in 1990 to more than six million by 2050 [5]. In Zimbabwe, hip fracture incidence rates for men and women aged 40 years and above was 46.6 and 58.8 per 100,000 people in 2022. Our work [6] has shown that, in older adults, hip fracture incidence rates for men and women in Zimbabwe exceeds those in Botswana and South Africa.

In South Africa, in the public hospital system, hip fractures are associated with a 33.5% one-year mortality [7,8], and both high direct (cost of treatment) and indirect (loss of income) economic impacts for the individual and their family [9]. Our work [10] found that the average total costs, in the first year post-hip fracture in South Africa, was US$6935 – 85% of which was attributable to an average 21-day hospital stay. Only a small body of research has investigated the financial burdens faced by orthopaedic trauma patients and their dependents during treatment and recovery in high-income countries, more so in an African setting [11]. Our recent economic analysis of hip fracture care in Zimbabwe showed that, although patients who received surgery for hip fractures faced substantially higher out-of-pocket (OOP) costs than those who were non-operatively managed, they had better health outcomes in terms of quality-adjusted life years (QALYs) [12].

Early operative treatment of hip fractures, within five days of presentation, is a globally-accepted standard of care, though this is challenging in low-resource-settings including in Zimbabwe [9,12–14]. In South Africa, orthopaedic emergencies are frequently delayed in favour of obstetric and surgical cases, resulting in a backlog of patients waiting an average of ten days for emergency surgery [15]. Streamlined, multidisciplinary management of hip fracture patients is one proposed approach for promoting timely surgery and improving patient outcomes. However, implementation is difficult in under-resourced low- and middle- income country (LMIC) health systems with typically siloed service delivery and an absence of clinical guidelines and protocols for hip fracture care [9,16]. Moreover, for older patients, hip fractures are more likely to be managed with non-operative treatment due to both resource and system constraints, including few surgeons providing musculoskeletal trauma care, limited operating theatre capacity, and inefficient processes for optimising patients with comorbidities [9]. In this way, peoples’ experiences of hip fracture care in LMICs can be shaped, not only by the availability of resources, but also by the institutional and organisational arrangements and processes relating to hip fracture care provision. Work by Gartner et al. [17] and others [18] has shown that experiences of care can be shaped by patients’ social, cultural, and economic concerns. Our recent cohort study in Zimbabwe demonstrated that it was characteristics of deprivation, including lower household income that predicted non-surgical hip fracture management, rather than a patients clinical condition, meaning the patients’ ability to pay is the main factor determining care [16]. It seems likely that the long recorded hospital stays at least partly reflect the time taken for a patient’s family to source the necessary out-of-pocket costs for surgery [16].

Health systems responsiveness was proposed by the World Health Organization (WHO) in 2000 and comprises seven elements – dignity, autonomy, confidentiality, prompt attention, access to social networks, quality of basic amenities, and choice of provider [19]. Subsequent frameworks, such as those by Valentine et al. [20] and Mirzoev and Kane [21] have suggested other domains of responsiveness, including trust and clarity of communication. Mirzoev and Kane [21], building upon existing conceptualisations, have argued that responsiveness entails the “actual experience of people’s interaction with their health system”. Such an experience, they suggest, is defined both by peoples’ expectations of care (shaped by intersecting social structures and relationships with family and community), and by the health system’s response to these needs and expectations (including, interactions with health professionals, managers, policymakers, and institutional and organisational arrangements, structures, and resources) [21].

Currently, peoples’ experiences of their interaction with health systems, with respect to responsiveness, has primarily been assessed through the WHO health systems responsiveness survey [22–25]. For instance, work by Peltzer [23] and others [26] used data from the Global Study on Aging and Adult Health (SAGE) to evaluate health systems responsiveness for older adults in South Africa. Patients’ greatest concerns related to long wait times at healthcare facilities, limited involvement in decision making, and poor communication from health professionals. A study by Muzvondiwa et al. [27] on the responsiveness of Zimbabwe’s health system to patients experiencing non-communicable diseases found escalating costs and scarcity of medications contributed towards patients losing trust in the health system. We recently analysed the availability of hip fracture services in The Gambia and Zimbabwe, identifying frequent non-operative management of hip fractures with extended delays for surgery, exacerbated by very low provision of all cadres of healthcare staff [28]. There is therefore an urgent need to understand to what extent health systems in Southern Africa are responsive to the needs of hip fracture patients. Such insights are vital to inform efforts aiming to strengthen health systems to meet changing needs, especially in response to rapid population ageing.

In this ethnographic study, we bridge the gap in available evidence on patients’ experiences of hip fracture care by examining the responsiveness of such care in Zimbabwe and the patient- and system-related factors influencing the provision of responsive care. Based on our findings, we outline key short-, medium-, and long-term recommendations, targeting each domain of responsiveness, for health system actors to consider to improve responsive hip fracture care in Zimbabwe, and similar health systems. We also discuss the implications of these findings on conceptualisations of health system responsiveness.

## Methods

### Study design

This ethnographic study was conducted as part of a wider mixed-methods research programme investigating the epidemiology, economic impact, and treatment of fractures and musculoskeletal health in The Gambia, South Africa, and Zimbabwe; the protocol has previously been published [29]

Ethnographic methods were used to understand the experiences of service users and characterise the delivery of hip fracture healthcare services [30]. The study was conducted in two sequential stages to allow findings from the earlier phase to inform subsequent inquiry:

Phase 1: Ethnographic case studies with patients and caregivers to understand experiences and views of hip fracture care.

Phase 2: Interviews with healthcare professionals and observations of practices to understand factors that help or hinder the implementation of hip fracture treatment.

This approach enabled us to first identify the priorities and experiences of patients and caregivers and then explore these issues from the perspective of healthcare professionals. This enabled us to understand what factors in the healthcare system contributed to the challenges and priorities identified by patient.

### Study settings

Within Zimbabwe’s decentralised health system, health care is provided at primary, secondary, tertiary, and quaternary levels [31,32]. Primary care, comprised of rural and urban clinics, is intended to be the entry-point for patients into the health system. Depending on the complexity of the health condition, patients may be referred to district and rural hospitals (secondary level) or for more specialised care and treatment to provincial (tertiary level) and central (quaternary level) hospitals [31,32]. Economic crises have resulted in a health system that is underfunded and strained by low health worker density, shortages of essential resources, the high burden of communicable and non-communicable diseases, and low health insurance coverage [31]. This has meant that the health system depends on user fees for revenue which, in turn, results in high out-of-pocket (OOP) health expenses for patients [31].

There are an estimated 2.2 facilities per 100,000 adults available for hip fracture presentation, with 69.4% of facilities (across care levels and including faith-based care) able to provide surgical services [29]. Severe cases such as orthopaedic trauma cases are further referred to central hospitals. These facilities have greater availability of specialists and clinicians, advanced equipment and pharmaceuticals, and act as the highest level for health referrals [31,32]. Five healthcare facilities providing hip fracture care were purposively sampled to represent a range of characteristics, e.g., facility type, service workloads, and geography of patient catchment areas.

### Participant recruitment

Phase 1 recruitment and data collection were conducted from May to December 2023. Patients were eligible for inclusion if they had experienced a hip fracture and were 40 years or older, this was to enable focus on older adults with fragility fractures. Hip fractures were categorised as “high-energy” if resulting from significant force, such as a car accident. “Low-energy” hip fractures were those resulting from minimal force, such as a fall from standing height or less, often suggesting underlying osteoporosis [33]. These were categorised based on the descriptions of the injury mechanisms described by patients and their caregivers. Caregivers were included anyone directly involved in the patient's care, e.g., family members, close friends and/or neighbours. Patients and caregivers were purposively sampled from five of the six healthcare facilities, considering age, sex, type of injury, comorbidities, and geographical location [34,35]. Potential participants were identified by reviewing hospital registers and emergency department records. Potential participants were approached in person by researchers in hospital, whilst those already discharged were visited at home after first being contacted by telephone. All 40 patients and 33 caregivers approached consented to participate. Patients with cognitive impairment were included to ensure equity and representation. Capacity to consent was assessed by trained research non-clinical personnel, in consultation with clinical colleagues where needed [36].

Phase 2 recruitment and data collection were conducted from June 2023 to March 2024. Health professionals who were involved in the delivery and organisation of hip fracture care were purposively sampled from across the care pathway, from six healthcare facilities [35]. Maximum variation sampling was used to include health professionals with a range of roles, and experience. Healthcare professionals were recruited by NB and TC. In total, 42 of the 43 healthcare providers approached agreed to participate in the study. Recruitment was influenced by the availability of healthcare professionals, as most interviews were conducted during working hours. Some participants were identified and approached based on observations made during Phase 1 data collection, prior to the start of recruitment for Phase 2. Additionally, snowball sampling was used as several healthcare professionals referred colleagues to the research team, enabling access to participants who might not have been easily reachable.

Sample size was determined by the achievement of information power which was assessed throughout data collection by reviewing collected data to identify gaps and identify areas requiring further exploration [37]. Through this iterative process, we judged the sample size to be sufficient when the dataset contained rich, detailed information that provided an in-depth understanding of the phenomena, addressed our research aims and yielded novel insights.

### Data collection

Phase 1 data collection was conducted using longitudinal ethnographic case studies, comprising repeated in-depth interviews with patients and their caregivers, complemented by observations of their home environments and care provision over an 8–10 month period [38]. Single or dyad interviews included persons who had experienced a hip fracture, along with their caregivers. Topic guides informed data collection and explored experiences and views of hip fracture treatment (S1 to S4 Guide). The number and timing of follow-up interviews was not influenced by whether participants chose to take part in individual or dyad interviews, but was shaped by methodological and practical considerations. Between one and three follow-up interviews (30–60 minutes each) were conducted, typically four to eight weeks after the initial interview. In terms of methodological considerations, participants were recruited at different points in their care journeys. For instance, some were identified during hospital admission prior to surgery, while others were recruited post-surgery. Care pathways also varied, with some participants accessing services such as physiotherapy while others did not. Follow-up interviews were therefore scheduled at key stages within each participant’s individual care journey rather than at fixed timepoints. Practical considerations also influenced the number and timing of interviews. Scheduling depended on participant availability, which was strongly influenced by their health status, as many participants were clinically vulnerable or unwell. In some cases, deterioration in health made additional interviews impossible, and, unfortunately, two participants died. Due to these circumstances, 19 patients were interviewed once, 12 were interviewed twice and seven three times.

All interviews were carried out in the local language of Shona by two researchers (NB and PM) in healthcare facilities or the homes of patients and caregivers. Interviews were audio recorded. In the event a patient felt uncomfortable speaking about their hospital experiences, the audio recording was paused, and notes were taken instead. Whether interviews were conducted individually or as dyad interviews was determined by participants preference and the involvement of family members or others in the patient’s care. Some patients chose to be interviewed individually which enabled them to reflect on their personal experiences, while others preferred to be interviewed alongside a caregiver who had been closely involved in their care. Dyad interviews with caregivers enabled shared recollection of events and clarification of experiences. Of a total of 105 interviews, 73 were single interviews and 32 were dyads.

Observation sessions in homes and hospitals helped understand patients’ social context. In Phase 1, observations of patients and caregivers were carried out in the hospital setting for patients who were recruited while admitted and in home settings. Observations of patients and caregivers were undertaken alongside interviews and informal discussions with the patient and family members who were present. In Phase 2, the researchers carried out observations of routine clinical practice and activities relating to hip fracture care. To guide data collection, observation checklists were devised to explore and describe settings, treatment practices and caregiving activities (S1 Checklist and S2 Checklist); fieldnotes were written to record observations. Approximately 22 hours of observations were conducted with patients and their caregivers.

To provide depth and context to Phase 1, Phase 2 data collection described decisions about care and barriers and facilitators to implementation of healthcare. Interviews with healthcare professional involved in the organisation and delivery of hip fracture care, explored care delivery throughout the care pathway, including emergency and orthopaedic care. Topic guides were used as above (S2 Guide). Interviews lasted between 16–95 minutes, and were conducted in Shona or English (depending on participants’ preferences) in healthcare facilities by two researchers (NB and TC). Researchers’ immersion in these contexts enabled them to assess the setting, as well as capture the non-verbal and verbal behaviours of healthcare professionals, patients and caregivers and their interactions. Observation checklists were used and fieldnotes written to record observations (S1 Checklist and S2 Checklist) and the informal conversations researchers conducted to clarify and make sense of what they were observing. Approximately 85 hours of observations with healthcare professionals were carried out.

### Data analysis

Analysis was iterative informing further participant recruitment and data collection [30]. Concurrent data collection and analysis was therefore important for determining final sample through ‘information power’ [37]. Analytical memos or short reflective notes were recorded by the study team in fieldwork diaries, summarising themes identified and outlined areas for further enquiry. The study team comprised of social scientists, Zimbabwean researchers, and healthcare professionals (including those working in Zimbabwe). Reflective note-taking enabled the research team to practice self-reflexivity – to consider the impact that their positionality had on their interactions with study participants [39]. The team held collaborative meetings as needed to discuss ideas and incorporate them into data collection tools [30]. During these collaborative meetings, the study team also reflected on how their personal and professional backgrounds may have shaped their interpretation of the data. All interviews, whether audio-recorded or noted, were carefully translated from Shona to English by the Zimbabwean-based researchers who understood the local context (NB, TC, and PM), transcribed verbatim, and anonymised to protect participants’ identities. Observations were written up into full fieldnotes. Data analysis followed three stages:

In stage one, data from interviews with patients and caregivers and observations with patients and caregivers were analysed by inductively coding transcripts to develop an initial analytical framework. The initial codes and themes were then applied to subsequent transcripts using NVivo qualitative analysis software [40]. Three researchers were involved in this process (NB, PM and SD) which included double coding and co-working to interpret data.

In stage two, analysis used an abductive analytical approach which, according to Tavory and Timmermans [41], involves a process of ‘theoretical sensitisation’ during which researchers familiarise themselves with theoretical literature pertinent to the inquiry. The iterative development of themes and sub-themes are therefore informed both by the data and theoretical literature on responsiveness. In response to insights identified from the data, the researchers consulted with theoretical literature on health systems responsiveness, including key works by de Silva [42], Valentine et al. [20], and Mirzoev and Kane [21]. Rather than double coding, validity in stage two was established through members of the research team comparing themes and subthemes identified (NB and SD). Codes and themes identified in the first stage were subsequently refined with reference to these theories.

In stage three, interview data from healthcare professionals and observations of hip fracture care provision in the study hospitals were imported into NVivo and analysed using an abductive approach identify the system-level factors that influenced the provision of responsive hip fracture care and refine existing sub-themes developed in stages 1 and 2.

### Ethics statement

Local ethical approval was provided by Biomedical Research and Training Institute (reference: AP161/2021) and the Medical Research Council of Zimbabwe and the Research Council of Zimbabwe (reference: MRCZ/A/2706). Administrative approvals and permission were sought from all hospitals where data were collected. The University of Bristol was the study sponsor (reference: NHE 05 CA 06 0013). Participants provided written consent and could provide additional consent for researchers to take photographs. Where potential participants lacked decisional capacity, consultees were approached to provide assent using the same processes. Two patients were assented through consultees.

## Results

In total, 40 patients were recruited (Table 1); 22 were male and 18 female. Most were identified and provided consent in hospital (n = 29), and only 11 at home following discharge. Male participants were younger than females; the female patients were more likely to have experienced a low energy fragility fracture consistent with osteoporosis. Only 55% males and 44% female received an operation for their hip fracture during the study period. Amongst males, 32% were living with HIV. Multimorbidity was common among females. The 42 healthcare professionals (54.7% female and 45.2% male) participating in the study spanned 12 different professional cadres (Table 2). The majority (52.4%) of healthcare professionals recruited had between one to nine years professional experience (Table 2). Table 3 shows that the majority of the 33 caregivers were female (82%), with the largest proportion aged 40-49 years (42%). Regarding their relationship to the parents, most caregivers were spouses (42%), followed by daughters (24%).

**Table 1:** Clinical and surgical characteristics of patients with hip fracture (results shown as percentages).

Characteristics		Male N = 22 N (%)	Female N = 18 N (%)
Age (years)	40-49	5 (22.7)	1 (5.6)
	50-59	9 (40.9)	1 (5.6)
	60-69	3 (13.6)	5 (27.8)
	70-79	3 (13.6)	8 (44.4)
	80 and above	2 (9.1)	3 (16.7)
Mechanism of injury	Low energy trauma	13 (59.1)	17 (94.4)
	High energy trauma	9 (40.9)	1 (5.6)
Surgical management	Operated	12 (54.5)	8 (44.4)
	Non-operated	7 (31.8)	2 (11.1)
	Awaiting operation	3 (13.6)	8 (44.4)
Co-morbidities	Living with HIV only	7 (31.8)	1 (5.6)
	Other only	3 (13.6)	5 (27.8)
	No reported co-morbidities	11 (50)	6 (33.3)
Multi-morbidities	Individuals with 2 or more co-morbidities	1 (4.5)	6 (33.3)

“High-energy trauma” – hip fractures resulting from significant force, such as a car accident

“Low-energy trauma” – hip fractures resulting from minimal force, such a fall from standing height or less

**Table 2:** Characteristics of healthcare professionals participating in the study.

Healthcare Professional Characteristics			Total N = 42 N (%)
Sex	Female		23 (54.7)
	Male		19 (45.2)
Age	20-29		5 (11.9)
	30-39		20 (47.6)
	40-49		10 (23.8)
	50-59		6 (14.3)
	Not disclosed		1 (2.4)
Professional role	Orthopaedic surgeon		6 (14.3)
	Theatre nurse		3 (7.1)
	Orthopaedic nurse		4 (9.5)
	Casualty nurse		2 (4.7)
	Casualty officer		2 (4.7)
	Matron		1 (2.4)
	Sister-in-charge		3 (7.1)
	Radiographer		4 (9.5)
	Physician		2 (4.7)
	Ward nurse		6 (14.3)
	Physiotherapist		4 (9.5)
	Occupational therapist		2 (4.7)
	Rehabilitation technician		1 (2.4)
	Pharmacist		2 (4.7)
Years of experience	1-9		22 (52.4)
	10-19		4 (9.5)
	20 and above		2 (4.7)
	Not disclosed		14 (33.3)

**Table 3:** Characteristics of caregivers participating in the study.

Caregiver/Caregiver Characteristics		Total N = 33 N (%)
Sex	Female	27 (81.8)
	Male	6 (18.2)
Age (years)	20-29	5 (15.2)
	30-39	4 (12.1)
	40-49	14 (42.4)
	50-59	3 (9.1)
	60-69	5 (15.2)
	70-79	2 (6.1)
Relationship to patient	Spouse	14 (42.4)
	Daughter	8 (24.2)
	Son	2 (6.1)
	Parent	2 (6.1)
	Granddaughter	1 (3.0)
	Sister	1 (3.0)
	Nephew	2 (6.1)
	Other	3 (9.1)

Below, we describe the six main thematic areas, with reference to key literature on health systems responsiveness. These themes were derived from an initial inductive analysis of interviews with patients and caregivers (stage 1), complimented by an abductive analysis of data collected from both healthcare professionals and patients and caregivers, along with conceptual literature on health systems responsiveness (stages 2 and 3). Patients, caregivers, and healthcare professionals highlighted needs and constraints relating to prompt provision of treatment, clear communication, dignified care, and social support. Perspectives revealed the importance of access to pain relief medication, as well as the impact of unaffordability of treatment, on their care experiences, these elements are distinct from those within current responsiveness frameworks. These findings have been presented in Fig 1.

**Fig 1:**
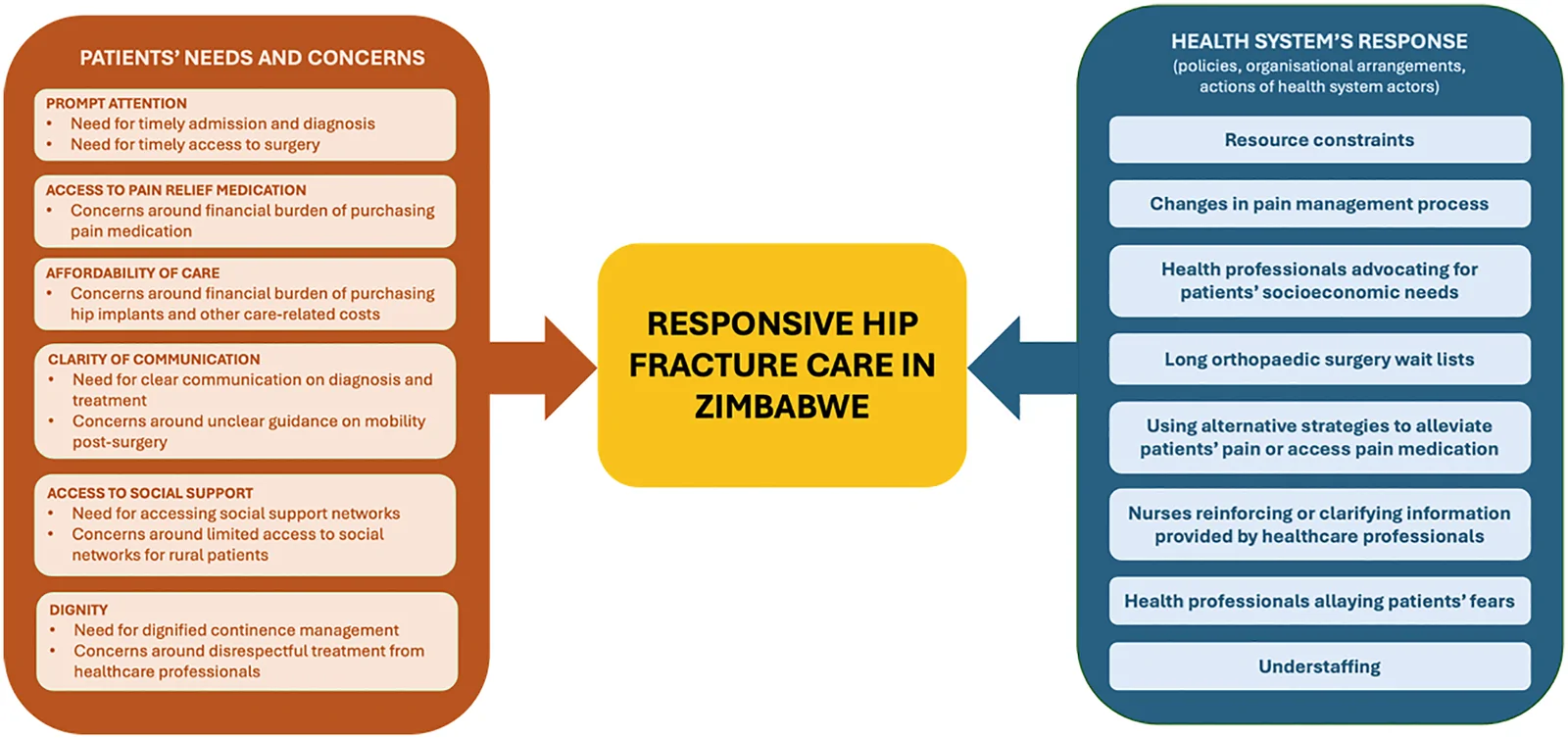
Experiences of responsive hip fracture care in Zimbabwe.

### Prompt attention

#### The need for timely admission and diagnosis.

According to de Silva [42], prompt attention within health systems responsiveness, corresponds to patients receiving care within reasonable time periods, avoiding long wait times for consultation and treatment, and rapid care in emergencies. Yet patients experiencing a hip fracture expressed frustration when describing the time it took to transition from the emergency department to the ward, due to extended wait times and administrative procedures. Lengthy queues of up to five hours for admission were common at the larger central hospitals. Being in pain only amplified patients’ distress in such situations:

*“I came with an ambulance from the accident scene…you will be in pain, they made me wait by the side, waiting for the doctor. At that time, I requested them, ‘I am in pain people, may I please have some painkillers’, then I was given an injection. I waited for a long time…”* ***Patient 4, 51-year-old man (Site 04).***

Once seen by the treating casualty officer (who is a medical doctor), patients with suspected hip fractures were referred for an x-ray to facilitate diagnosis and treatment. Patients experienced extended delays and notes from an observation vividly describes the emotions and interactions at one such x-ray department:

*“In the waiting area of the x-ray department, the behaviours and interactions between patients and those who had accompanied them was a mix of patience, frustration, and occasional camaraderie…The patients frequently expressed their impatience, especially when one patient entered the x-ray room and took longer than expected. There were murmurs of discontent about the wait times, with some patients audibly wondering aloud why it's taking so long.”* ***Observation notes (Site 04)***

During this observation, the researcher noted the strain the long wait placed on older patients in particular, who were seen falling asleep and with limited access to food and water. The nurse on duty later explained that, despite efforts to prioritise certain groups, such as older patients, there were factors beyond their control affecting waiting times. In this way, interviews with health professionals highlighted the pressures they experienced which, at times, prevented them from responding to patients’ needs and expectations of prompt attention. As one health professional explained, these could relate to unforeseen issues with x-ray machines, including no electricity and unmet maintenance requirements:

*“…The x-ray machines require maintenance, from time to time, they break down, if they do breakdown, then they would have to go and get an x-ray done somewhere else”* ***Surgeon (Site 03).***

The “somewhere else” was a private health facility, though health professionals pointed out that accessibility of such facilities were an issue due to a lack of transportation. Electricity outages also meant that lifts in the hospital no longer worked, making it difficult to transfer patients to departments on different floors, including to theatre for surgery.

#### The need for timely access to hip fracture surgery.

Another need communicated by patients and their caregivers was timeliness to hip fracture surgery, as also outline in Fig 1. They shared experiences of extended hospital stays due to repeated postponements of their surgeries caused by over-running theatre lists, as well as the cost of implants (described further below):

*“My surgery has been postponed twice, my family had finally bought the implants I finally asked one of the nurses why is it this happening and she was honest and told me that there is a long theatre list…”* ***Patient 1, 49-year-old man (Site 04)***

Health professionals were acutely aware of these pressures and described taking steps to advocate for patients’ needs for timely surgery, including lobbying for a dedicated list for hip fracture surgeries:

*“What we are trying to lobby at the moment is to get a dedicated trauma list [for hip fracture], that is there so that at least you have got two operating chances per week.”* ***Surgeon, (Site 03).***

Adding to the discomfort and emotional strain of a lengthy hospital stay, was being placed ‘nil by mouth’ before surgery and not being able to eat or drink for an extended period. As one patient explains, limited communication from health professionals added to the uncertainty:

*“I have been told you are going to theatre tomorrow, then it ends up being 3 or 4 days, and they will be telling you daily, you are not eating today, you are going to theatre. The last time they said one guys operation took long and so I could not be operated, and I had not eaten all day, they should at least tell you on time, isn’t it?”* ***Patient 2, 52-year-old man (Site 03).***

Health professionals’ perspectives revealed how certain procedures and processes complicated providing timely access to hip fracture surgery, including standard procedures for optimising patients with co-morbidities:

*“…but they are various factors which may cause delays in surgery, some of them which are quite common are co-morbidities because many of these particularly the elderly people they have got various co-morbidities, the need to be optimised before they can be taken for surgery, so that alone can actually contribute to delay in surgery.”* ***Surgeon (Site 03).***

The length of time taken to optimise patients, but also for families to purchase implants, often resulted in the development of pressure sores (bedsores). Patients described painful pressure sores persisting for weeks, further extending the timeline to surgery:

*“I have had these bedsores for two weeks now and I have been in hospital since the 2*^*nd*^ *of November (about three weeks ago). They had asked us to purchase the implants and we looked for the money so I could be in theatre on the 9*^*th*^ *of November, but I didn’t’ go and we just waited, then they [family] asked why the surgery has not been done, when we have bought everything…and they said we cannot because the bedsores have not dried up…I asked again today because I am in pain and they said if we go to operate it may cause other diseases [infection] … They want the bedsores to really dry up….”* ***Patient 15, Site 03***

In line with best practice, healthcare professionals thought that patients should be turned every two hours though, due to staff shortages, health professionals explained that this was not always feasible. Additionally, as the quotation below illustrates, another significant constraint to timely surgery were the limited operating times in government hospitals, resulting in long wait lists for surgeries. Contributing to the backlog of patients, were shortages of surgical instruments, anaesthetic drugs and, in some facilities, there was only one trained orthopaedic surgeon.

*“Then also in government [hospitals] we have a problem of operating time, so we only…like for example we operate once a week, if someone is admitted let’s say on a Thursday, even if we optimise them with 48 hours, they still have to wait a couple of days for their turn…for the operating date to come, so the operating time is also a major factor there.”* ***Surgeon (Site 03).***

*“...there is only one surgeon in [Provincial hospital]; he can be overwhelmed with patients. We have only one.... sometimes he might not finish all the patients depending with the type of the surgery that must be done.” Physiotherapist (Site 05).*

Health professionals also mentioned that theatre lists for hip fracture surgeries were impacted by emergency cases, including ‘open fractures’ (where there is an open wound) which were prioritised for surgery over hip fractures:

*“...open fractures, they are emergency, they must be operated within 24 hours, so it means these are open wounds, they are the ones…the first priority, it means our list is going down.”* ***Orthopaedic nurse (Site 04).***

As outlined in Fig 1, prompt access to treatment and surgery for hip fractures was clearly an important need and concern for patients and their caregivers. However, health professional accounts shed light on several health system constraints and pressures (some of which were beyond health professionals’ control) that restricted their efforts to respond to these needs.

### Access to pain relief medication

#### Changes in drug procurement procedures.

Distinct from the domain of prompt attention, patients stressed the importance of timely access to pain relief medication when first arriving at a health care facility with a hip fracture. As the quote below highlights and as shown in Fig 1, patients experienced severe pain, but were hampered by the lengthy administrative processes from receiving prompt pain medication:

*“I was in severe pain and I was sitting on a wheelchair that my nephew had found in the hospital…we waited for some time, if they had given me something for the pain while all the forms were being filled in, it would have been helpful…I don’t want to lie to you my child it was painful when I broke this leg…”* ***Patient 10, 69-year-old man (Site 03).***

Health professionals explained how changes to drug procurement systems in public hospitals (due to wider governmental transitions) underpinned the limited availability of pain relief medication. As outlined below, health professionals clarified that while Pethidine (an opioid pain medication) is still stocked in hospitals, other pain relief medications (such as paracetamol) now needed to be purchased over-the-counter.

*“…from the new era yes [when there was a transition in the government]. That’s when things just started to deteriorate. We used to have our medication, ya we used to have. The paracet, the tablets, even that tramadol, the hospital used to supply”* ***Casualty nurse (Site 05).***

#### The financial burden of purchasing pain medication.

In some instances, health professionals requested that family members purchase (with their own funds) pain relief medication from a private pharmacy, outside the hospital. This meant that patients often relied on family members to obtain and deliver their medication, leaving them in pain while waiting for the medication to be bought. Patients and their families (especially those from rural areas) also faced a financial burden of purchasing, and travelling to purchase, pain relief medication (as noted in Fig 1):

*“I feel for her [my wife] she has to come here every day to see if there are any medication or anything that is needed to be bought…she did not come one day because she had no money for transport and I did not have any pills, my leg was paining badly but I did not get any medication…you can die from pain here, they must do something…”* ***Patient 9, 51-year-old man (Site 03)***.

Nevertheless, health professionals were cognisant of the need to improve pain management systems and employed several strategies to alleviate patients’ pain, including using linen to align the leg, cold and warm compresses to reduce swelling, providing other forms of pain relief medication (like paracetamol or morphine) or using the medication of other patients (those who had been discharged or had passed away):

*“Some whom would have been discharged who are well up, can leave some pills for you and say please keep and give to those who won’t have, or even those with their relatives who would have passed away can just leave the pills.”* ***Nurse-in-charge (Site 03).***

Health professionals mentioned that the urgency to provide pain relief stemmed from the emotional toll of witnessing patients in pain:

*“There are times when you see a patient who has a fracture and is in a lot of pain that you can really see that they are in pain. Yes, we try to wear the nurse’s face of being strong…”* ***Registered nurse (Site 02).***

Therefore, though both patients and health professionals emphasised the importance of receiving pain relief medication, it was clear that timely access was hampered by changes to pain management procedures which, in turn, placed additional financial burdens on patients and their families.

### Affordability of care

#### The financial burden of purchasing hip implants and other care-related costs.

The affordability of responsive care, and the subsequent impact on one’s socioeconomic situation, is not sufficiently acknowledged within current responsiveness frameworks and resultant literature. Yet, both patients and their caregivers explained to us how their socioeconomic condition determined whether (or not) the hip fracture care they received responded to their needs and priorities. As we have outlined above, patients stressed the importance of receiving hip fracture surgery in a timely manner. The inability to afford implants for hip surgery, however, often meant that some patients remained in hospital and awaited surgery until their family could raise the necessary funds. Paradoxically, the long hospital stays ultimately increased their in-patient hospital bill. The cost of implants could range from $500 all the way up to $1500 US dollars, with 63.8% of individuals in the study setting reporting a household income of <100USD per month. Notes from an observation thus reveal how some patients were discharged due to the mounting in-patient hospital bill.

*“An 84-year-old individual, had been in the ward for over two months and was due to be discharged on that day. However, the circumstances surrounding the discharge were quite poignant. Despite the need for surgery, the patient and his family members couldn't afford to purchase the required implants, leading to the decision to get him discharged. Moreover, due to the mounting hospital bills resulting from the prolonged stay, the patient's relatives had made the difficult choice to request discharge. As I was in the ward the patient was sleeping all the times and looked frail and not fit for discharge.”* ***Observation notes (Site 03).***

It also became apparent that it was not only implants that needed to be purchased. Patients reported facing additional expenses for essential medical supplies including, traction kits, bandages, as well as anticoagulant and pain relief medications (as we have examined above). Costs associated with transportation, both in relation to seeking care and for transferring the patient between facilities, presented an additional financial obstacle. The carer’s account below illustrates how the high costs of private transportation can often result in the injured patient remaining at home until funds can be found – sometimes by borrowing and pooling funds from friends and relatives.

*“We did not go the day it happened because we were looking for money. We needed money to take her from here with a private car. It was not possible to use a commuter omnibus, so we looked to hire a private car to take her to the hospital. Some charge 30 or 25 US dollars, to drop you off then pick you up, so we asked our son who is going to school, to borrow money so we can go to the hospital. He ran around and borrowed from his friends, and that is the money we used to go to the hospital…”* ***Carer 12, 58-year-old man (Site 03).***

Transportation costs for attending physiotherapy sessions or outpatient orthopaedic appointments added further financial pressure, especially for patients residing in rural areas. Given this, in some instances, patients and their families reported having to make difficult decisions, weighing up between meeting their healthcare needs and maintaining basic living necessities. Experiencing a hip fracture therefore had a broader impact on the household economy, especially if the patient was the primary income provider for the family:

*“My injury has affected my life…yes. The work that I was doing as a man [stammers] you will be doing odd jobs so that you can live [survive], now when it is like that how do you live [survive]. How do you live…isn’t it the family is looking to you…”* ***Patient 2, 74-year-old man (Site 02)***

#### Health professionals advocating for patients’ socioeconomic needs.

Interviews with health professionals revealed the various strategies and practices they employed to enable hip fracture patients to receive the care they needed, despite their inability to pay. This involved looking to the patients’ social support system, for instance:

*“…we also try to establish the support system at home, who are the people who are taking care of the patient at home, and the social circumstances of the patient, right [speaks in Shona] is the patient a destitute …such patients for example, they will never be able to…to afford these implants, those are some of the patients we intervene.”* ***Orthopaedic surgeon (Site 05).***

The “intervention” the orthopaedic surgeon alludes to here was referral to the social welfare department who could facilitate payment of hospital bills. Leveraging the existing public-private partnerships formed with implant companies was another strategy health professionals utilised to mitigate high treatment costs. Health professionals described instances of advocating on behalf of low-income patients – requesting free or highly subsidised implants from these companies (see under ‘health system’s response’ on Fig 1):

*“…we tend to establish a good relationship with different implant companies, such that in special cases really, we talk to these guys, they give us the implants in special cases…For free, or for highly subsidised.”* ***Orthopaedic surgeon (Site 05).***

The above evidence illustrates that patients’ ability to receive hip fracture care, in a timely manner, was also, in part, influenced by the affordability of such care – a key element we outline in Fig 1. That said, health professional accounts expose the several courses of action they pursued to overcome these constraints leading to some exceptional cases where hip fracture patients received timely care and treatment.

### Clarity of communication

#### The need for clear communication on diagnosis and treatment.

In a subsequent conceptualisation of responsiveness by Valentine and colleagues [20], “clarity of communication” corresponds to health professionals clearly explaining the nature of a patient’s illness, including details of the treatment and options. In the narratives of hip fracture patients were clear concerns about not having regular interactions with their assigned doctors once admitted into the ward. As such, some patients spoke about lacking clarity on their diagnosis and treatment plan. As the quotation below suggests, physicians were pressed for time and not able to explain the treatment plan to patients in detail:

*“They can go for three days without coming and when they come, they have no time to fully explain to you. They might have a lot of patients, so when they pass by my bed, they just say we will come on Thursday, and this will be on Tuesday…”* ***Patient 8, 41-year-old man (Site 03).***

In their definition of “clarity of communication”, Valentine et al. [20] emphasise the importance of treatment plans and options being clearly communicated to both patients and their families. Yet, some caregivers pointed out that they did not get the opportunity to obtain information on diagnosis or treatment as the doctor’s rounds and the visiting hours did not coincide:

*“I can say that we never spoke to the doctors. Isn’t they do their rounds when we are not there. In the case of today it’s only because they wanted pictures. But if there are any other things needed or medications needed, we are just told when we come in the afternoon what will be written. We don’t see the doctors...”* ***Carer 2, 29-year-old woman (Site 03)***.

#### Concerns around unclear guidance on mobility post-surgery.

Upon discharge, and as noted in Fig 1, patients and caregivers expressed concerns about inconsistent or unclear guidance regarding mobility from different members of the care team. Some reported not receiving any specific instructions on how to mobilise post-surgery, while others received conflicting advice. For instance, in one case, the patient was advised to start weightbearing activities while caregivers were told that weight should not be placed on the hip. Speaking with health professionals exposed the various health system constraints impacting their ability to provide clear communication to hip fracture patients. One challenge, as the nurse below describes, was understaffing:

*“One orthopaedic surgeon is not enough l believe that our surgeon is overwhelmed, l think we need two so that they alternate-because currently our surgeon is managing male ward, female ward, children’s ward, OPD, sometimes Rehab so for one person it’s not working at all.”* ***Nurse (Site 05).***

Due to these constraints, nurses explained how they acted as a bridge between health professionals and patients – reinforcing and/or clarifying the information provided by health professionals on treatment and discharge. Despite their high workload, some nurses also mentioned taking the time to sit with patients to allay their fears and anxieties, especially with regards to fears of being permanently disabled after surgery (see under ‘health system’s response’ in Fig 1):

*“…they will be anxious whether [they] will be going to walk, so basically what we will tell [them] to allay [their] anxiety, we will tell [them] you are hurt and it will take time to heal…So that information will be told the patient starting from the casualty so that the patient will not feel like he/she is going to be disabled.”* ***Nurse (Site 05).***

While receiving clear and timely communication on diagnosis and treatment was an apparent need expressed by both patients and their caregivers, we observed again how health system-related pressures became obstacles for health professionals working to meet these needs.

### Access to social support

#### The need for accessing social support networks.

De Silva [42] proposes that the provision of responsive, in-patient health care should include regular visits by one’s relatives and friends, including the provision of food and other essential items by such individuals. Observations illustrated the importance of visits by family members for providing the patient with food from home, clean clothes, as well as medications which were required to be purchased from private pharmacies. Overall, visits from family members and friends contributed towards the emotional well-being of patients. During one observation, the researcher noted the evident change in atmosphere on the ward following the end of visiting hours:

*“I couldn't help but notice the change in atmosphere as the visitors departed…. Some patients wore sullen expressions, perhaps feeling the absence of relatives and visitors…As I left the female ward, I couldn't help but feel a sense of sadness after having observed the patients change in mood after their visitors left.”* ***Observation notes (Site 05).***

It was also noticed during the observations that some patients did not receive any visitors. Upon seeking clarification from the nurse-in-charge, the researcher identified the impact that a patients’ rural location can have on access to their social support network (as outline in Fig 1):

*“She [nurse-in-charge] elaborated by pointing out that the provincial hospital serves a wide catchment area, leading to some patients residing in rural or distant town far from the facility. As a result, it may not be feasible for their relatives to make daily trips to visit them due to the associated travel costs”* ***Observation notes (Site 05).***

#### Limited access to social networks for rural patients.

Health care facilities did not have the capacity to accommodate relatives. For those from rural areas, relatives went so far as to rent a room in town to remain close to the patient and to avoid travel expenses – particularly in the event their hospital stay was prolonged. In such cases, patients became a source of advice and encouragement to other patients during the lengthy hospital stay. Forming such connections was also the means through which some patients bridged gaps in information:

*“Information would come from the next bed; you would hear from your neighbour. Your neighbour is the one who would tell you, ‘My friend, for you to be operated on soon, why don’t you do this and this’. Then when my wife comes, I tell her we must do such and such, like the others…”* ***Patient 4, 47-year-old man (Site 04).***

As the nurse describes below, some older hip fracture patients experienced depression because of the social isolation that came with a lengthy hospital stay – especially if they could not be visited by their relatives. Patients therefore appreciated the emotional support that fellow patients could offer.

*“Most of them they get into depression, they are old...Sometimes you can hear someone saying, “I want to go outside in the sun”, but their leg is tied down. So sometimes they end up getting depressed. You see grandfather crying on his bed, and you try and talk to them and they say I want to go home.”* ***Nurse (Site 02).***

The support of one’s social network was just as vital following discharge when it came to receiving physiotherapy following discharge from hip fracture surgery. As one researcher noted during an observation, support from family can facilitate a patient’s rehabilitation, especially for older individuals

*“…physiotherapist permitted the carers to accompany them into the treatment room. This not only provided emotional support to the patients but also allowed carers to be directly involved in the rehabilitation process. I overheard one particular interaction that underscored the importance of allowing the carers to accompany them in the treatment room. A carer was encouraging her elderly relative, who was over 65, to stay strong and continue walking on the treadmill [which was recently donated]* **.” *Observation notes (Site 04).***

Social support is an important element of patients’ hip fracture care and recovery. Though their experiences revealed that access to such support was shaped by intersecting forces relating to both their socioeconomic situation, as well as their rural location. But also, by health care facilities’ capacity to accommodate patients’ social support needs.

### Dignity

#### The need for dignified continence management.

Dignified care, as defined within responsiveness, acknowledges a patient’s right to privacy during the course of their treatment [42]. The interviews and observations exposed instances of patients’ privacy being breached. This was primarily in relation to the use of bed pans – the general consensus amongst patients was that bed pans came at scheduled times, and outside of this, they were unable to request for them. Some patients reported not receiving bed pans on time which meant they soiled themselves, leading to considerable discomfort and embarrassment (a ‘need’ outlined in Fig 1):

*“Some of the nurses here they are not good. You will call out and they will ignore, or they will tell you it is not time for bed pans. It is not because we enjoy using the bed pans, but I am not able to walk. No one in here enjoys calling out for a bed pan but you have no choice. Sometimes you end up just going because they are late in bringing the bed pans, and they get mad with you when you soil yourself…it is not good”* ***Patient 6, 50-year-old man (Site 04)***.

Feeling hesitant and embarrassed, some patients shared that they often avoided eating meals to prevent the need to request for a bed pan. Observations revealed that, in certain hospitals, there were no curtains or screens in place to ensure patient privacy. Hence, it was possible to see patients changing their clothes or using bedpans. This was even the case in mixed (male and female) wards:

*“Here as you can see, we are mixed [male and female], we are all sick, but we just see each other [no privacy] …”* ***Patient 9, 46-year-old man (Site 04).***

Health professionals confirmed that bed pans were given according to a schedule to create a routine for patients, while enabling nurses to manage their tasks. Though, as this nurse explains, the absence of curtains and screens meant that bed pans were not offered during mealtimes or visiting hours:

*“…l am speaking for the female ward, bed pan is given per request except if its visiting hour or during feeds, so for our feeds porridge is given at 6a.m, tea time its 9-10, lunch time its 12:00-13:00, supper its 16:00-17:00 so during those time you can’t request for a bed pan because you cannot do your business here and the next patient is eating, we don’t have patterns or cubic so there is no privacy, even the screens we only have one screen and also to avoid the odour, you cannot give someone the bed when the other one is eating but besides that we offer bed pans”* ***Nurse (Site 05).***

That said, some nurses mentioned taking the time to offer patients reassurance and allay their concerns about using bedpans around other patients. Other nurses (such as the nurse below) described using particularly innovative approaches to maintain patients’ privacy:

*“We don’t have screens so if 2 patients ask for a bed pan it means only one of them is going to get privacy, we have got only one screen in the ward, the other is not going to get the privacy especially if they can’t wait, and the screen is not even the actual screen [laughing], the screens are improvised, like the ones we have now its umm just the combination of bars then we just fastened a fleece blanket around and its just one in this whole ward so you can just imagine.”* ***Nurse (Site 05).***

#### Concerns around disrespectful treatment from healthcare professionals.

Another aspect of dignified care, within responsiveness, is being treated with respect by healthcare staff [42]. Some patients felt that health professionals lacked empathy and were rude and unresponsive to their needs. Health professionals, on the other hand, explained how being overworked due to staff shortages can mean they are sometimes (unintentionally) disrespectful to patients:

*“...l think usually its due to shortage of staff...usually you become rude when you are very tired, anything that needs you, that needs your attention, l admit healthcare workers tend to be rude, but sometimes its unintentional so usually its just being overwhelmed by work that lead to being rude sometimes or just ignore, so the patient will feel umm this one is rude....patients will not understand what is on the ground”* ***Theatre nurse (Site 05).***

The provision of dignified care is a key feature of responsiveness. In their narratives, hip fracture patients conveyed the distress and embarrassment they experienced when their privacy was compromised or when treated disrespectfully by healthcare staff. Health professionals’ perspectives further demonstrated how their ability to respond to patients’ dignity-related needs was hampered by resource constraints – a key aspect of the ‘health system’s response’ we highlight in Fig 1.

## Discussion

This ethnographic study examined the responsiveness of hip fracture care in Zimbabwe. By collecting and analysing the narratives of patients, caregivers, and health professionals, as well as taking and examining fieldnotes from observations of healthcare facilities and participants’ homes, we have identified the various health system- and patient-related forces that shape experiences of hip fracture care. These findings are summarised in Fig 1.

On arrival at a health facility, hip fracture patients and their caregivers experienced complicated administrative procedures and long wait times for x-rays, delaying diagnosis and treatment. Health professionals explained that a lack of electricity and malfunctioning equipment – issues beyond their control – prevented them from providing timely attention and treatment. This included non-operational lifts during electricity outages which prevented transfer of patients from the ward (which was on a different floor) to theatre for surgery. Pouramin et al.’s work [43] echoes this finding – they identified that, in LMICs (namely, China, Africa, India, south and east Asia, and Latin America), patients with hip fractures risk delays of more than 24 hours due to deficiencies in x-ray facilities. For some patients, their need to access timely hip fracture surgery was also not met. While this was partly due to over-running theatre lists, patients’ inability to pay for implants greatly prolonged their hospital stays. A study examining the determinants of access to orthopaedic trauma surgery in Uganda identified social capital – the leveraging of one’s social network – as the strongest determinant of access [44]. Indeed, in our study, patients and caregivers accounts revealed how funds were often borrowed from friends and family not only for purchasing implants, (which could range from $500 to $1500 US dollars) but also for costs related to transportation. Hence, OOP costs associated with hip fracture care can be catastrophic – especially when taking into account that 63.8% of study individuals reported a household income of <100USD per month [45]. Access to social support is an important element of responsive care and the observations and interviews illustrated hip fracture patients’ need for such support during their hospital stay, which included the provision of food and other essentials and the raising of funds for implants. The support of one’s social network was also important post-discharge for rehabilitation. That said, we found that patients’ rural location often presented a barrier to accessing their social network and resulted in additional financial burdens. This is a concern for many - approximately half of the patients seeking hip care in the six hospitals came from rural areas [13].

Evidence on the management of acute pain from trauma, including musculoskeletal-related trauma, is currently lacking in LMICs, despite resource and system challenges. Studies in Nigeria, for example, have revealed how governmental restrictions on opioid dispensing and high OOP health expenditure can be persistent barriers to accessing pain relief medication for marginalised groups [46]. In a similar way, in this study, patients described being in extreme pain, with provision of certain pain relief medication being restricted by now longstanding changes to the drug procurement system in public hospitals. Patients therefore relied on caregivers to purchase such medication presenting further financial challenges, especially for those in rural areas. Patients and caregivers also expressed frustration at receiving inconsistent information, on diagnosis and treatment, from different members of the team, perhaps reflecting a lack of multidisciplinary teamwork. There was also conflicting guidance from health professionals on mobility post-discharge – in line with standard practice, patients with fragility fractures should be allowed to fully bear weight the day after surgery [13]. Standard guidance such as this can allow for clearer communication on mobility post-discharge for patients and their caregivers (see recommendation in Table 4). Patients expressed a clear need for dignified continence management – providing bed pans when needed and with privacy. As these needs were often not met, some patients explained avoiding food and drink. Not knowing when and, and indeed if, their hip fracture surgery would take place also saw patients being placed ‘nil by mouth’ for an extended period. This cycle of undernutrition can predispose patients to pressure sores – further prolonging time to surgery. Nutrition is a key pillar of hip fracture care, with standard guidance specifying the need to minimise the duration of pre-operative fasting [13]. This underscores the importance of smooth communication in the event of surgery cancellation/ postponement to allow patients to receive some nutrition.

**Table 4:** Recommendations for improving the responsiveness of hip fracture care in Zimbabwe.

Recommendations for improving the responsiveness of hip fracture care in Zimbabwe		Domain of responsiveness					
**Prompt attention**	**Access to pain relief medication**	**Affordability** **of care**	**Clarity** **of communication**	**Access to social support**	**Dignity**
**Short-term**	**Train HCPs to provide information to patients and caregivers, in a respectful manner on administrative processes, cancellation of operations to allow resumption of eating, diagnosis and treatment plans, as well as post-operative mobilisation and rehabilitation**						
	**Turn/reposition hip fracture patients every two hours, in line with best practice**						
	**Administer pain relief medication immediately for suspected hip fractures prior to x-ray and admission to wards**						
	**Designate nurses as point-of-contact for patients to reinforce health information and treatment plans from doctors**						
	**Provide communal/faith-based activities in wards to increase social support to inpatients**						
	**Give guidance to nurses on the provision of bedpans outside scheduled times**						
	**Reassure patients to allay discomfort and fears on using bedpans**						
	**Provide curtains/screens in wards to maintain patient privacy**						
	**Create age-friendly environments in hospital waiting areas (provide chairs, rails, wheelchair accessibility)**						
**Medium-term**	**Prioritise suspected hip fractures for x-ray to reduce wait time**						
	**Prioritise hip fractures on theatre lists**						
	**Maintain audit of essential surgical equipment and anaesthetic drugs**						
	**Standardise costs of implants to reduce variation in prices**						
	**Establish formal agreement between public hospitals and private implant companies to source implants at reduced costs and free/subsidised for patients with very low incomes**						
	**Standardised guidance and train healthcare professionals, including nurses, on how to mobilise patients post-surgery**						
	**Routinely provide information to patients and caregivers on rehabilitation, including verbal AND written information**						
	**Develop guidelines on the optimisation of patients for surgery which outlines conditions requiring optimisation, investigations needed, to reduce timelines for optimisation**						
	**Develop Zimbabwean guidelines on hip fracture management, including recommendations to provide surgery within a maximum of five days from admission**						
	**Maintain a stock of pain relief medication in hospital pharmacies**						
	**Simplify administrative process to expedite admission from emergency department/ orthopaedic clinics to wards**						
**Long-term**	**Increase the number of trained orthopaedic surgeons**						
	**Increase orthopaedic surgical capacity at district-level facilities**						
	**Develop & routinely provide community physiotherapy to patients post discharge**						

Health professionals’ narratives highlighted the various actions and practices they employed to circumvent health system pressures and constraints and respond to patients’ needs. While some wards lacked screens or curtains, health professionals described constructing their own to maintain patients’ privacy and dignity, especially with the use of bedpans. Health professionals were cognisant of the financial barriers patients faced when seeking hip fracture care and advocated on their behalf to the social welfare department and even to private firms supplying hip implants. In this way, while several health system-related pressures and processes clearly hampered the provision of responsive hip fracture care, our findings have shed light on the actions health professionals employed to overcome these barriers and respond to patients’ needs.

Our findings therefore have specific implications for understanding and examining health systems responsiveness, especially in resource-constrained settings. The affordability of hip fracture care was a primary concern for patients in our study. This was unsurprising given that previous work has highlighted both the direct and indirect costs associated with hip fracture care and treatment [9]. Indeed, our own cohort study has shown that non-surgical treatment of hip fractures was associated with factors such as the ability to pay [12]. These findings were confirmed in our study as patients explained how their inability to afford implants (and other essential medical supplies) extended their timeline to surgery while, at the same time, increasing their in-patient hospital bill. Their need for accessing timely treatment, a key consideration within the notion of health systems responsiveness, was thus often not met due to their poverty. Patients and their families also spoke about the financial burden associated with purchasing pain relief medication and attending physiotherapy sessions or outpatient orthopaedic appointments, which was further complicated by some patient’s rural location. It was therefore evident that receiving responsive hip fracture care was closely dependent upon whether or not patients were able to pay for the associated services. Especially as insurance coverage remains low across Zimbabwe [12].

As Mirzoev and Kane [21] have argued, there is a need for conceptualisations of responsiveness, and resultant empirical literature, to engage with the socio-cultural-economic context within which health system interactions occur – a consideration yet to be captured within current responsiveness frameworks. More recent work [47] has emphasised the importance of considering how socioeconomic concerns shape responsive care provision. User fees were introduced in Zimbabwe in the early 1990s in line with the Economic Structural Adjustment Programme (ESAP) and has become an important source of revenue for the public health system, given poor government investment [31]. However, a user exemption policy does remain in place for specific groups, including patients above 65 years. That being the case, we observed that older hip fracture patients still had to bear the burden of purchasing implants (with costs varying between private implant firms), essential medical supplies (especially pain relief medication), and costs associated with admission and transportation. The inability to afford such costs meant the hip fracture care most patients received ultimately did not respond to their needs. Considering our findings, we suggest that future research should acknowledge how socioeconomic concerns shape patients’ ability to pay for and ultimately receive responsive care. We echo the recent calls [47] for an expanded conceptualisation of responsiveness – one which acknowledges how socioeconomic forces shape the health system’s response (policies, organisational arrangements, as well as the actions and interactions of health system actors) to people’s expectations of care. But also, how socioeconomic concerns shape patients’ needs and expectations, especially regarding orthopaedic care.

Patients and caregivers highlighted several expectations, needs, and concerns relating to hip fracture care. Clarity of communication by health professionals was a priority indicated by patients and caregivers, and this element of responsive care has been acknowledged by Valentine and colleagues [20]. Another need was timely access to pain relief medication – an aspect of care that does not align with the WHO-proposed responsiveness domains which centre on the ‘non-medical’ aspects of care [19]. Nevertheless, access to pain relief medication was a key expectation raised by patients in this study which could not be ignored. The elements of care deemed important by patients and caregivers, we suggest, could reflect the health condition for which care is sought, as well as patients’ and caregivers’ social and economic circumstances. Further research is thus needed to characterise patients’ expectations of hip fracture care, beyond the WHO-proposed domains. The interconnected nature of the elements of care patients and caregivers discussed was also clear – they appeared to reinforce or counter each other. For instance, being able to afford implants impacted patients’ ability to receive timely surgery. While, prompt surgery and a shorter hospital stay, in turn, reduced inpatient hospital bills. It is therefore apparent that the elements of health systems responsiveness should not be analysed in isolation from one another, but rather as intersecting, interconnected domains. We propose that such an analysis can allow researchers to shed light on the intricacies of patients’ care experiences.

### Recommendations

Based on our findings, we outline key short-, medium-, and long-term recommendations that health system actors can consider for improving the responsiveness of hip fracture care in Zimbabwe. We define short-term recommendations as those that can be implemented now, with little adjustments needed to existing procedures and structures. We believe these initiatives can have an immediate impact on improving the responsiveness the hip fracture care. Medium-term actions are those which would require collaborations between health professionals, facility managers, and/or policymakers to change or modify certain hip fracture care-related processes and activities. Finally, long-term recommendations are defined as those requiring extensive collaborations between health system actors, at all levels, over an extended period. As we highlight below in Table 4, many of these actions and initiatives can address several elements of responsive hip fracture care concurrently.

### Strengths and limitations

Using an ethnographic longitudinal case study approach allowed us to conduct an in-depth exploration of patients’ experiences of hip fracture care, as well as health professionals’ perceptions on the pressures and constraints influencing responsive hip fracture care provision. Work was carried out by the same researchers during Phases 1 and 2 of data collection. This was advantageous as it enabled the researchers to develop a deep understanding of the issues under study and develop relationships to support data collection. This helped to yield rich data and to ensure that insights from the first phase were used to inform the focus of subsequent inquiry. Observations also enabled researchers to situate themselves within patients’ social contexts, as well as the context of care provision, to capture the complexity of the hip fracture care experience – which may not be fully understood through only conducting in-depth interviews. Purposive sampling allowed us to recruit a diversity of service users regarding age, sex, commodities, and geographical location which was important for shedding light on the various patient-related forces (such as their socioeconomic condition) which shaped their care experiences.

The 8–10-month fieldwork phases was a strength of the study since it enabled us to carry out longitudinal case studies with patients and caregivers, including follow-up interviews 4–8 weeks after the initial interview to capture experiences of hip fracture care at different stages of the care pathway. The timeframe also enabled us to recruit patients and caregivers from sites with lower volumes of hip fracture patients, particularly district hospitals with fewer hip fracture cases. It also provided flexibility to accommodate interviews with busy healthcare professionals from across the hip fracture care pathway. In addition, it provided us with the scope to conduct preliminary analysis alongside data collection, supporting iterative sampling and refinement of data collection tools. In this way, the duration of data collection periods strengthened the depth and rigour of the study.

Moreover, the response rate for patients and healthcare professionals in this study was very high (100%). This may partly reflect the inclusion of patients who had previously participated in other work packages within the wider Fractures E3 study [29], which may have increased their willingness to take part. Fewer caregivers were recruited than patients because we typically approached caregivers in person during home visits, meaning participation depended on their availability at that moment. While this method helped us recruit those most involved in daily care, it also meant that some caregiver groups may have been overlooked. Among healthcare professionals, high participation may have been facilitated by the rapport established during Phase 1 of the study and the flexibility offered in scheduling interviews, including choice of time and venue.

We recognise that there is potential that participants may modify their behaviour in response to the researcher’s presence. While this cannot be fully eliminated, we sought to mitigate its effects and account for it analytically. We prioritised building rapport and trust with healthcare professionals, including clarifying the researcher’s role as a non-judgemental observer to reduce the perception amongst participants that they were being evaluated. The use of multiple data sources (observations and interviews) enabled us to identify and interrogate discrepancies between observed practices and participant accounts. In addition, we also engaged in reflective practice throughout the study, with the research team regularly considering how the researcher’s presence may have shaped interactions and behaviours, informing data interpretation. Despite these measures, some degree of reactivity is likely to have persisted and influenced observed behaviours.

Tavory and Timmermans [41] have (see S1 to S4 Guide and S1 and S2 Checklist) suggested that an abductive analytical approach allows researchers to develop novel theorisations by identifying evidence which lies outside the study’s theoretical frame. In this way, while our analysis was informed by theoretical literature on responsiveness, themes and sub-themes were also iteratively defined by insights emerging from the data – such as those relating to patients’ access to pain relief medication. This iterative approach also prompted us to consider health professionals’ perspectives of hip fracture care provision in the second phase of data analysis. Therefore, we have been able to highlight the actions and strategies health professionals employed to overcome health system pressures and constraints. Data analysis was also conducted by a research team with expertise in the study context (NB, PM, and TC), as well as with the methodologies employed (SD, RGH, KL). However, that being said, health professionals may have been reluctant to share instances of poor practice observed within the health facilities they worked, and which may have influenced patients’ reported experiences of hip fracture care.

## Conclusion

As populations in Southern Africa age, health systems must adapt to respond to changing needs. Hip fractures have major health and economic consequences. Multidisciplinary management can promote timely surgery and improve patient outcomes but is difficult to implement in under-resourced health systems. Until now, no study had examined the extent to which health systems, globally, are responsive to hip fracture patients’ needs, including the impact of health system-related pressures and constraints. In this ethnographic study, we have addressed this gap by revealing hip fracture patients’ and caregivers’ needs relating to timely and dignified provision of care, clear communication from health professionals, and the support of their social networks. Distinct from the domains of responsiveness, patients and caregivers also stressed the importance of accessing pain relief medication, and affordable care. Through the narratives of health professionals, we have shown how understaffing, resource constraints, and long surgical delays impact the health system’s ability to meet to patients’ needs. Yet we uncovered how health professionals employed various strategies to overcome these barriers and respond to patient priorities. Our findings have facilitated the development of practical recommendations for health system actors to consider improving responsive hip fracture care in Zimbabwe.

## Supporting information

S1_Guide Topic Guide Patients. (DOCX)

S2_Guide Topic Guide Family and Carers. (DOCX)

S3_Guide Topic Guide Health Care Workers – Surgeons & Theatre Nurses. (DOCX)

S4_Guide Topic Guide Health Care Workers – Non Surgeons. (DOCX)

S1_Checklist Observation Schedule Healthcare Facilities. (DOC)

S2_Checklist Observation Schedule Community Settings. (DOC)
