# Supplementary material for: Examining the patient- and system-related forces influencing the responsiveness of hip fracture care in Zimbabwe: Findings from an ethnographic study
Source: PLOS Glob Public Health. 2026 Nov 4;6(11):e0006631. doi: 10.1371/journal.pgph.0006631 (PMC13734578; doi:10.1371/journal.pgph.0006631)
Supplement: Supplementary file 1 [file pgph.0006631.s001.docx]

**Fractures in Sub-Saharan Africa – The Fractures E3 Study**

**A study of hip fracture care**

**TOPIC GUIDE PATIENTS**

Interviews will focus on the topics below and will be informed by observational fieldwork.

**Aims and objectives**

- To understand what it is like to break a hip and their views and experiences of accessing care.
- This study will provide us with information that can be used to improve the care people receive when they have broken a hip.

**Introduction**

- Introduce self
- Introduce the study: who is it for, what is it about
- Key points - Length of interview, voluntary nature of participation and right to withdraw, recording of the interview
- Confidentiality and how findings will be reported - No names, published in reports and academic publications, short quotes from them in write-up
- Any questions that they have
- Take consent – signed consent, thumb print (with witness), verbal (audio recorded)

**Part 1: Find out a bit about you**

- Tell me about yourself
- Age
- Sex
- What level of education do you have
- What do you do on a daily basis? If retired, what did you do before?
- Where do you live? (Urban/ rural location)
- How far away do you live from the hospital where you are now?
- What’s your marital status? (married/single/ spouse alive or dead)
- Who do you live with?
- Did you have another illness before you broke your hip What was it?
- Have you broken a bone before?

**Part 2: Life before you broke your hip**

- What did you do before you broke your hip (working, caring for other people, e.g. grandchildren)?
- What did you do for fun?
- What was life like before you broke your hip?

**Part 3: Experiences of breaking hip**

- Tell me about experiences of a breaking hip
  - When did it happen (date and time)?
  - How did it happen?
  - Who were you with?
  - How did it feel?
  - What did you think had happened?
  - What did you do?

**Part 4: Accessing treatment**

- Tell me about your experience of getting treatment for your broken hip
  - When did you decide to get treatment?
  - What made you decide to get treatment?
  - Did you speak to anyone about it?
  - Did you try any other treatments before you went to clinic/ hospital (care for it themselves, traditional practices)?
  - Where did you decide to go and get treatment (e.g. did they do to a local clinic/ central hospital)? Why?
  - How long did you wait until you decided to get treatment at a clinic/ hospital?
  - What transport did you use?
  - Was it your own transport or was it hired (how much?)
  - How long did you wait to get help?
  - What was the journey like?
- Was there anything that made it difficult for you to get treatment at the hospital/ clinic? (e.g. long distance from services, cost of transport, other responsibilities)
- Was there anything that helped make it easier for you to get treatment at the hospital/ clinic? (e.g. relatives living nearby, help with transport costs)

**Part 5: Experiences/ views of treatment**

- What happened to you when you first visited hospital/ clinic? (NB: Encourage patients to explain what happened step-by-step)
- Who did you speak to when you arrived?
- What happened next?
- What treatment did you receive?
- How did you find out you had broken your hip? (e.g. x-ray, told by doctor)
- Were you in pain? If so, was anything done to make the pain better?
- Did you have any other symptoms that were troubling you? (e.g. incontinence) If so, was anything done to make them better?
- Have you visited/ been told to go to any other hospital or service? (e.g. from smaller district hospital to central hospital). If so:
  - Which hospital/ clinic?
  - Why (e.g. no operating theatre)?
  - How did you get there?
  - What was the journey like?
  - What happened when you got there?
- Which healthcare professionals have been involved in caring for you?
  - What do each of them do?
- What is your communication (or relationship?) like with the healthcare professionals caring for you?
  - What have they told you?
  - Do you feel like you understand everything that is happening?
  - Anything missing?
- Did you have an operation?
- Why/ why not?
- Who made this decision?
- How do you feel about being in hospital?
  - Is there anything that worries you (e.g. caring responsibilities, finances)?
  - Anything that makes you feel better?
- Is there anything you think could have been done to improve your time in hospital?
- What do you expect to happen next in your treatment?

**Part 6: Recovery**

- What happened when you were discharged from hospital?
  - How was this done?
  - Where you given information on how to care for yourself?
  - Who gave you this information?
  - How did you get home?
- How do you feel about no longer being in hospital?
- Since you have been back home, how have you been doing?
- What is a normal day like for you now?
- How much of a problem is your injury?
  - What do you find difficult? (e.g. pain)
  - What do you do to try and manage this? (e.g. painkillers, rest)
- What is different about your life compared to just before your injury?
  - What has made the most difference to your life?
- Compared to just before your injury, what has stayed the same?
- Have you made any changes to your life to make your recovery easier (e.g. moved to be closer to relatives, made adaptations to home)?
- How useful has this been?
- How did you make this decision?
- Is there anyone who is helping to look after you?
  - What do they do?
  - How useful is this?
- How have you been managing financially? (Probe on how they are prioritizing)
- What has been difficult? (e.g. paying for treatment, no longer in employment)
- Which Family Member?
- How did they (you) afford this? [probes: savings, selling things, sacrifice other items]
- How have you been managing this? (hospital bills)
- Is there anything else you would find useful to help you recover?
  - Why?
- What are your relationships with other people like since you have broken your hip (e.g: burden to others, etc.)
- What have people around you said about your injury? (e.g. neighbours, those in the community)
- Do you have any follow-up appointments planned with healthcare services?
  - What are these?
  - What will happen?
  - How do you feel about the follow-up visits? Is it helpful?
- Since returning home, have you looked anywhere else for help? (e.g. bought painkillers, traditional and religious)
- Is there anything that worries you? What? (e.g. finances)

**Part 7: Close**

- Any other insights/ issues to discuss
- Questions about the project
- Thank them
- Reaffirm confidentiality
