# Supplementary material for: Examining the patient- and system-related forces influencing the responsiveness of hip fracture care in Zimbabwe: Findings from an ethnographic study
Source: PLOS Glob Public Health. 2026 Nov 4;6(11):e0006631. doi: 10.1371/journal.pgph.0006631 (PMC13734578; doi:10.1371/journal.pgph.0006631)
Supplement: Supplementary file 2 [file pgph.0006631.s002.docx]

**Fractures in Sub-Saharan Africa – The Fractures E3 Study**

**A study of hip fracture care**

**TOPIC GUIDE FAMILY AND CARERS**

Interviews will focus on the topics below and will be informed by observational fieldwork.

**Aims and objectives**

- To understand what it is like to break a hip and your views and experiences of their care.
- This study will provide us with information that can be used to improve the care people receive when they have broken a hip.

**Introduction**

- Introduce self
- Introduce the study: who is it for, what is it about
- Key points - Length of interview, voluntary nature of participation and right to withdraw, recording of the interview
- Confidentiality and how findings will be reported - No names, published in reports and academic publications, short quotes from them in write-up
- Any questions that they have
- Take consent – signed consent, thumb print (with witness), verbal (audio recorded)

**Part 1: Find out a bit about you**

- Tell me about yourself
- Age
- Sex
- What level of education do you have
- What do you do on a daily basis? If retired, what did you do before?
- Have you always lived here?
- What’s your marital status? (married/single/ spouse alive or dead)
- Who do you live with?
- Do you have any illness?
- Have you broken a bone before?

**Part 2: Life before they broke their hip**

- What did they do before they broke their hip (working, caring for other people, e.g. grandchildren)?
- What was your life like before they broke their hip?
- How has your life changed, if at all?

**Part 3: Experiences of breaking hip**

- Tell me about their experiences of a breaking hip
- Were you there when it happened?
  - When did it happen (date and time)?
  - How did it happen?
  - Who were they with?
  - Who told you?
  - What did you think had happened?
  - What did you do?

**Part 4: Accessing treatment**

- Tell me about what you know about their experience of getting treatment for their broken hip
  - When did they decide to get treatment?
  - Who decided for them to get that treatment?
  - What made them decide to get treatment?
  - Did they speak to anyone about it?
  - Did they get any other treatments before they went the clinic/ hospital (care for it themselves, traditional practices)?
  - Where did they decide to go and get treatment (e.g. did they go to a local clinic/ central hospital)?
  - How long did they wait until they decided to get treatment at a clinic/ hospital?
  - What transport did they use?
  - Was it their own transport or was it hired (how much?)
  - How long did they wait to get help?
  - What was the journey like?
  - Where you there from the beginning or you joined midway or at the end of this journey?
- Was there anything that made it difficult for them to get treatment at the hospital/ clinic? (e.g. long distance from services, cost of transport, other responsibilities)
- Was there anything that helped make it easier for them to get treatment at the hospital/ clinic? (e.g. relatives living nearby, help with transport costs)

**Part 5: Experiences/ views of treatment**

- What happened when they first visited hospital/ clinic? (If they accompanied them)
- Who did you speak to when you arrived?
- What happened next?
- What treatment did they receive?
- How did they find out they had broken their hip? (e.g. x-ray, told by doctor)
- Were they in pain? If so, was anything done to make the pain better?
- Did they have any other symptoms that were troubling them? If so, was anything done to make them better?
- Did they visit/ been told to go to any other hospital or service? (e.g. from smaller district hospital to central hospital). If so:
  - Which hospital/ clinic?
  - Why (e.g. no operating theatre)?
  - How did they get there?
  - What was the journey like?
  - What happened when they got there?
- Which healthcare professionals have been involved in caring for them?
  - What do each of them do?
- What is your communication (or relationship?) like with the healthcare professionals caring for them?
  - What have they told you?
  - Do you feel like you understand everything that is happening?
- Did they have an operation?
- Why/ why not?
- Who made this decision?

**Part 6: Recovery**

- What happened when they were discharged from hospital?
  - How was this done?
- Where you given information on how to care for them?
- Who gave you this information?
- How did they get home?
- How do you feel about them no longer being in hospital?
  - No longer doing hospital visits
- Since they have been back home, how have they been doing?
- What is their normal day like for them now?
- How much of a problem is their injury?
  - What do you find difficult? (e.g. caring for them, bathing, changing, etc)
  - What do you do to try and manage this? (e.g. getting extra help from other relatives)
- What is different about their life compared to just before your injury?
  - What has made the most difference to theirs?
- Compared to just before their injury, what has stayed the same?
- Have you made any changes to your life to make their recovery easier (e.g. changing work hours, made adaptations to home)?
- How useful has this been?
- How did you make this decision?
- Is there anyone who is helping you to look after them?
  - What do they do?
  - How useful is this?
- How are you managing with your finances? (Probe on how they are prioritizing)
- Is there anyone helping you financially?
- Have you cleared your hospital bills?
- Is there anything else you would find useful to help with their recover?
  - Why?
- What are your relationships with them or other family members like since they have broken their hip (e.g: strained relationship, etc.)
- Do they have any follow-up appointments planned with healthcare services that you are aware of?
  - What are these?
  - What will happen?
  - How do you feel about the follow-up visits? Is it helpful?
- Since returning home, have you or they been anywhere else for help? (e.g. bought painkillers, traditional and religious)
- What do you think will happen in the future?
- Is there anything that worries you? What? (e.g. finances)
- Is there anything that you are looking forward to?

**Part 7: Close**

- Any other insights/ issues to discuss
- Questions about the project
- Thank them
- Reaffirm confidentiality
