# Supplementary material for: Examining the patient- and system-related forces influencing the responsiveness of hip fracture care in Zimbabwe: Findings from an ethnographic study
Source: PLOS Glob Public Health. 2026 Nov 4;6(11):e0006631. doi: 10.1371/journal.pgph.0006631 (PMC13734578; doi:10.1371/journal.pgph.0006631)
Supplement: Supplementary file 3 [file pgph.0006631.s003.docx]

**Fractures in Sub-Saharan Africa – The Fractures E3 Study**

**A study of hip fracture care**

**TOPIC GUIDE HEALTH CARE WORKERS – SURGEONS & THEATRE NURSES**

Interviews will focus on the topics below and will be informed by observational fieldwork.

**Aims and objectives**

- To understand hip fracture service provision from a provider perspective, focusing on their views and experiences of providing services and of uptake of services by hip fracture clients.
- This study will provide us with information that can be used to improve the care people receive when they have broken a hip.

**Introduction**

- Introduce yourself
- Introduce the study: who is it for, and what is it about.
  - Key points – length of the interview, voluntary nature of participation, the right to withdraw, and audio recording of the interview.
  - Confidentiality and how findings will be reported – no names, published in reports and academic publications, short quotes from them in write-up
  - Any questions that they have
  - Take consent – signed consent, thumb print (with witness), verbal (audio-recorded)

**Part 1: Find out a bit about you**

1) May you please kindly tell me about yourself.
   - What is your job title? (ask for official post as per their contract)
   - Place(s) of work (public and private), how long have you worked in this position?
   - Age, gender, education level
   - Describe the department you work in, and what is done there in relation to hip fracture care pathway.
2) Please describe the department you work in, your day-to-day activities in relation to hip fracture care. [NB: if they do both public and private ask them to distinguish between the two].
   - Describe your working hours, and daily activities/assigned responsibilities, including your on call commitments (how often in the hospital vs at home)
   - How many hip fracture patients do you see a week in an average week?
   - Describe the diagnostic process (i.e how do you know it’s a hip fracture, and what diagnostic tests do you use? – i.e. X-rays, etc)
   - What alternatives are available when X-ray equipment is not working?

**Part 2: Your role in relation to hip fracture care**

3) Describe to me the leadership structure (power dynamics/lead persons/reporting structure) of hip fracture care in the health facility [compare public and private where if applicable].

- who makes decisions about patient care - to what extent are decisions shared, and do patients have the autonomy of the care they receive. - to what extent are the 4 principles (Do not harm, doing good, freedom of choice, ensuring fairness) of practicing medicine enforced
  - *** probe the following for surgeons only. Do you view yourself as a team leader? Please describe whether you believe other than the surgery that you are fully responsible for the patient’s complete hip fracture care pathway.

4) Describe to me how you feel about your job and the emotions triggered by your experiences in providing hip fracture care.

- Kindly explain why you chose this career path. What dreams, hopes and aspirations did you have? How do your dreams then relate to your experiences now – is it as you had imagined, explain?
- Describe the emotional feelings triggered from doing the procedures based on your desire to do what’s best for the client but fail to do so due to resource constraints.
- As part of phase 1, at times patients expressed the lack of energy or empathy from healthcare workers. How do you feel about this, do you believe this is a true reflection of healthcare worker attitudes.
- Describe to me some of the job-related frustrations that you encounter. How best do you feel this research can bring this out – please share any recommendations that you feel we should put forward that would help to make your work better.

**Part 3: Hip fracture pain management**

5) Please describe your perception of the pain experienced by hip fracture patients and explain the pain management the patient gets in practice.

- Where do patients first get pain medication?
  - What pain medication is provided, is it available? If pain medication not available at health facility, where do patients or carers purchase medication and how far are the pharmacies from the health facility.
  - Describe the frequency of pain management offered to patients.
  - Where pain medication is not available, are different measures taken when caring for the patient? If so, what?

6) Please share your own recommendations on how to improve pain management for patients. What structures need to be put in place for you to better manage patients’ pain?

**Part 4: Theatre/surgery [For surgeons and theatre nurses]**

7) Please describe the different hip fracture treatment options that are available at your facility?
8) Describe the factors that are considered when deciding whether to perform surgery or not.
   - How does the type of injury inform and affect treatment/surgery?
   - Describe how availability of resources affects getting surgery or not.
9) Thinking now about the factors that affect timeliness to surgery (from admission of the patient to time of surgery). Please give examples of cases where patients have had to wait different time frames, and
   - Explain some of the causes of delays to surgery. How does resource availability affect time to surgery (i.e., resources, surgeon/human resources, theatre availability)
   - To what extent are hip fracture patients prioritised when there are long queues for surgery? How does this prioritisation work in practice? If not prioritised, does gender or age play a role?
   - Describe how a patient’s general health (co-morbidities, bed sores, etc) can affect decisions to operate and timeliness to surgery
   - Do you collect any data on how your orthopaedic service is performing that you review as an ‘audit’? If so, is this done to see if you could improve care? Has it led to any changes?
10) During observations and discussion with patients, some get weighed, and others did not. What influences whether patients get weighted or not.
11) Kindly share your own recommendations on how best to improve timeliness to surgery considering the factors mentioned above.
12) Hip fracture care is provided by the combined effort of a number of health care professionals, how well do you think you work as a team? Can you give an example of what might improve the teamwork?

**Part 5: Hip fracture ward care**

13) Thinking now about the hospital post-operation/rehabilitation management for hip fracture patients.
    - Describe what happens post-op in hospital in the care of hip fracture patients. (e.g. do you visit patients on the ward? Do you instruct physiotherapy? Do you re-Xray patients?
    - What post operative complications do you see and what are the most serious?
    - On average how long after an operation is done is a patient discharged?
14) Considering the processes taken to help mobilize patients.
    - Describe the services provided, the frequency that they are provided (in reality) (Physiotherapy, Family support etc)
    - For patients who can’t afford private physiotherapy, describe your experiences in how patients are mobilised, if they are
    - Describe any walking aid resources availed to the patient. Are these free/purchased? (How much, If not purchased at hospital, how much it costs outside the hospital if they know)
15) Please describe the hospital discharge process.
    - Are patients discharged straight home or do they go elsewhere? (e.g. other hospital/clinic)
    - When do you follow up patients?
    - Do you give advice to patients and/or carers about what they should do post discharge? If so, what do you say? (e.g., mobilisation, pain management, home adaptation etc). Who communicates this, and how is it communicated?

**Part 6: Patient well-being [NB: Omit for theatre nurses]**

16) From your experience, describe the impact of hip fracture injuries on patients, and their carers. Do you find it differs by the age of the patient?

- Giving a few examples, describe the concerns, fears, and experiences of hip fracture patients.
  - Describe their financial and livelihood concerns.
  - Describe any fears of disability.
  - Do these concerns trouble you personally?
  - How comfortable do you feel talking to patients about their concerns around their hip fracture?

**Part 7: Financing Hip fracture treatment**

17) Describe some options available for ‘free healthcare’ for those with financial challenges.

- If there is some aid given, how do they determine who is eligible for this assistance, and who is involved in the decision making?
