# Supplementary material for: Examining the patient- and system-related forces influencing the responsiveness of hip fracture care in Zimbabwe: Findings from an ethnographic study
Source: PLOS Glob Public Health. 2026 Nov 4;6(11):e0006631. doi: 10.1371/journal.pgph.0006631 (PMC13734578; doi:10.1371/journal.pgph.0006631)
Supplement: Supplementary file 4 [file pgph.0006631.s004.docx]

**Fractures in Sub-Saharan Africa – The Fractures E3 Study**

**A study of hip fracture care**

**TOPIC GUIDE HEALTH CARE WORKERS – NON SURGEONS**

Interviews will focus on the topics below and will be informed by observational fieldwork.

**Aims and objectives**

- To understand hip fracture service provision from a provider perspective, focusing on their views and experiences of providing services and of uptake of services by hip fracture clients.
- This study will provide us with information that can be used to improve the care people receive when they have broken a hip.

**Introduction**

- Introduce yourself
- Introduce the study: who is it for, and what is it about.
  - Key points – length of the interview, voluntary nature of participation, the right to withdraw, and audio recording of the interview.
  - Confidentiality and how findings will be reported – no names, published in reports and academic publications, short quotes from them in write-up
  - Any questions that they have
  - Take consent – signed consent, thumb print (with witness), verbal (audio-recorded)

**Part 1: Find out a bit about you**

1) May you please kindly tell me about yourself.
   - What is your job title?
   - Place(s) of work
   - Age, gender, education level
2) Please describe your day to day activities at work, i.e. job description.
   - How long have you been working in this position?
   - Describe your work hours, and daily activities/assigned responsibilities
   - Do you enjoy your job?

**Part 2: Your job in relation to hip fracture care**

3) Thank you for sharing details about your work and what you do on a daily basis. Can you describe what hip fracture care available at this particular healthcare facility and which part of the care pathway that you provide.
   - Describe the services this facility offers from the patient reaching this facility. Organizatioinal structure particularly with hip fractures.
   - Where is the first point of care, and what treatment/services are offered first to the patient?
   - Describe the nurse patient ratio.
   - Which parts of that care do you particularly provide?
   - Describe the diagnostic process (i.e how do you know it’s a hip fracture, tests are performed? – i.e. x-rays, etc)
   - What do patients need, that can’t be provided in this facility?
   - How many hip fracture patients do you see on average in a week?
4) Describe to me how you feel about your job, and contribution towards hip-fracture care.
   - What emotional feelings are triggered in relation to you satisfying your ethical obligations as a healthcare worker.
   - Describe what triggers happy feelings, and frustrations within your line of work.
   - How best can this research shed light on your experiences, and what recommendations can we put forward that would help improve your job and ability to satisfy your ethical obligations to your patients.

**Part 3: Hip fracture pain management [Note: not to be asked to people in non-patient facing roles]**

5) Please describe your perception of the pain experienced by hip fracture patients and the emotions triggered within you when you see your clients going through the pain.
   - Would you say one gets used to seeing the patients in pain, describe your coping mechanisms?
6) Kindly explain the pain management process from the time the patient gets to the facility.
   - Where do patients first get pain medication?
   - What pain medication is provided, is it available? If pain medication not available at health facility, where do patients or carers purchase medication and how far are the pharmacies from the health facility.
   - Describe the frequency of pain management offered to patients.
   - Where pain medication is not available, are different measures taken when caring for the patient? If so, what?
7) Please share your own recommendations on how to improve pain management for patients. What structures need to be put in place for you to better manage patients’ pain?

**Part 4: Patient well-being (Phrase question to applicable to participants role)**

8) From your experience, describe the impact of hip fracture injuries on patients, and their carers. Do you find it differs by the age of the patient?
9) Giving a few examples, describe the concerns, fears, and experiences of hip fracture patients.
   - Describe their financial and livelihood concerns.
   - Describe any fears of disability.
   - Do these concerns trouble you personally?
   - How comfortable do you feel talking to patients about their concerns around their hip fracture?
10) Describe the culture of communication with hip fractures patients.
    - Describe the way you personally relate with patients. Describe the communication strategy you use. Do you take time to know them personally or you keep it professional.
    - Describe the way in which surgeons/doctors relate to patients. Who is responsible for communication with the patient about their hip fracture treatment?
11) **[Question for nursing staff, aides etc]** Hip fractures make patients immobile, so they need help to go to the toilet. Describe how patients are assisted with toilet services. Is that what you do?
    a. Describe the frequency of bed pans on the ward, who assists the patients, what is done to provide privacy?
    b. Describe the concerns patients have about this.
    c. Describe approaches implemented (if any) by hospital staff/patients to reduce toilet use.

**Part 5: Theatre/surgery**

12) Please describe the different hip fracture treatment options that are available at your facility (surgery and non surgical options)?
13) Thinking now about the factors that affect timeliness to surgery (from admission of the patient to time of surgery). Please give examples of cases where patients have had to wait different time frames, and
    a. Explain some of the causes of delays to surgery. How does resource availability affect time to surgery (i.e., resources, surgeon/human resources, theatre availability)
    b. To what extent are hip fracture patients prioritised when there are long queues for surgery? How does this prioritisation work in practice? If not prioritised, does gender or age play a role?
    c. Describe how a patient’s general health (co-morbidities, bed sores, etc) can affect decisions to operate and timeliness to surgery
    d. When there are delays in surgery, please describe the routine of patient care (eg. Repeated Nil by mouth rules from midnight to be followed, etc)
14) During observations and discussion with patients, some get weighed, and others did not. What influences whether patients get weighted or not.
15) Kindly share your own recommendations on how best to improve care of patients with hip fracture considering the factors mentioned above.
16) Hip fracture care is provided by the combined effort of a number of health care professionals, how well do you think you work as a team? Can you give an example of what might improve the team work?

**Part 6: Access to hip fracture ward care [NOTE: not to be asked non-ward HCW]**

17) Thinking now about the hospital post-operation/rehabilitation management for hip fracture patients.
    a. Describe what happens post-op in hospital in the care of hip fracture patients..
    b. What post operative complications do you see and what are the most serious?
    c. On average how long after an operation is done is a patient discharged?
18) Considering the processes taken to help mobilize patients.
    a. Describe the services provided, the frequency that they are provided (in reality) (Physiotherapy, Family support etc)
    b. For patients who can’t afford private physiotherapy, describe your experiences in how patients are mobilised, if they are
    c. Describe any walking aid resources availed to the patient. Are these free/purchased? (how much)
19) Please describe the hospital discharge process.
    a. Are patients discharged straight home or do they go elsewhere? (e.g. other hospital/clinic)
    b. When do you follow up patients?
    c. Do you give advice to patients and/or carers about what they should do post discharge? If so, what do you say? (e.g., mobilisation, pain management, home adaptation etc).

**Part 7: Financing Hip fracture treatment [Note: only asked to those with administrative roles (sic, administrator, physiotherapist, x-ray department]**

20) Describe some options available for ‘free healthcare’ for those with financial challenges.

- If there is some aid given, how do they determine who is eligible for this assistance, and who is involved in the decision making?
