# Supplementary material for: Examining the patient- and system-related forces influencing the responsiveness of hip fracture care in Zimbabwe: Findings from an ethnographic study
Source: PLOS Glob Public Health. 2026 Nov 4;6(11):e0006631. doi: 10.1371/journal.pgph.0006631 (PMC13734578; doi:10.1371/journal.pgph.0006631)
Supplement: Supplementary file 5 [file pgph.0006631.s005.doc]

For office use only: to be completed by research staff

| Site | 0 |  |
| --- | --- | --- |

**Fractures in Sub-Saharan Africa – The Fractures E3 Study**

**A study of hip fracture care**

**OBSERVATION SCHEDULE HEALTHCARE FACILITIES**

**Details of observation session:**

| **Location:** |  |
| --- | --- |
| **Time and date:** |  |
| **Observation number:** |  |
| **Length of time spent observing:**  **Person carrying out observation:** |  |

| **Number of patients and carers**  **Description of patients and carers** | **Number of healthcare professionals**  **Description of healthcare professionals, including types** |
| --- | --- |
|  |  |
| **Description of setting, including layout of facility** | |
|  | |
| **Description of treatment being delivered** | |
|  | |
| **Description of behaviour of patients and carers, including conversations taking place** | |
|  | |
| **Description of interactions between healthcare professionals and patients, including conversations** | |
|  | |
| **Description of interactions between healthcare professionals, including conversations** | |
|  | |
| **Identification of what works well about the service** | |
|  | |
| **Identification of challenges within the service** | |
|  | |
| **General thoughts and comments** | |
|  | |
