# Supplementary material for: Examining the patient- and system-related forces influencing the responsiveness of hip fracture care in Zimbabwe: Findings from an ethnographic study
Source: PLOS Glob Public Health. 2026 Nov 4;6(11):e0006631. doi: 10.1371/journal.pgph.0006631 (PMC13734578; doi:10.1371/journal.pgph.0006631)
Supplement: Supplementary file 6 [file pgph.0006631.s006.doc]

For office use only: to be completed by research staff

| Site |  |  |  | Participant ID |  |  |  |
| --- | --- | --- | --- | --- | --- | --- | --- |

**FRACTURES-E3: A study of hip fracture care**

**OBSERVATION SCHEDULE COMMUNITY SETTINGS**

**Details of observation session:**

| **Location:** |  |
| --- | --- |
| **Time and date:** |  |
| **Observation number:** |  |
| **Length of time spent observing:** |  |

**Topics to explore:**

- Description of participants, e.g. wealth, age, mobility
- Description of setting, including accessibility, adaptations to environment
- Activities taking place, including caring responsibilities
- Interactions between family members/ people around them
